# Adjuvant Chemotherapy and Chemoradiotherapy in Gastric Cancer: Prognostic Determinants and Real-World Survival Outcomes

**DOI:** 10.3390/jcm15020553

**Published:** 2026-01-09

**Authors:** Sedat Yildirim, Hatice Odabas, Seval Ay Ersoy, Seval Orman, Miray Aydogan, Ezgi Turkoglu, Goncagul Akdag, Hamit Bal, Melike Pekyurek Varan, Deniz Isik, Nedim Turan

**Affiliations:** 1 Department of Medical Oncology, Kartal Dr. Lütfi Kirdar City Hospital, Health Science University, 34865 Istanbul, Türkiye; odabashatice@yahoo.com (H.O.); drsevalay@gmail.com (S.A.E.); drseval1988@gmail.com (S.O.); mirayaydogan1991@gmail.com (M.A.); ezgiturk_90@hotmail.com (E.T.); akdaggoncagul@gmail.com (G.A.); hamitbal@yahoo.com (H.B.); dnz.1984@yahoo.com (D.I.); turan.nedim@hotmail.com (N.T.); 2 Department of Radiation Oncology, Kartal Dr. Lütfi Kirdar City Hospital, Health Science University, 34865 Istanbul, Türkiye; melikepekyurek@gmail.com

**Keywords:** adenocarcinoma, chemoradiotherapy, gastrectomy, gastric cancer, adjuvant

## Abstract

**Objective:** The role of adjuvant chemoradiotherapy (CRT) following curative gastrectomy remains controversial, especially in the context of D2 dissection. This research evaluated survival indicators through an analysis of previous observational studies and it evaluated treatment outcomes between patients who underwent CRT and those who received CT as their sole therapy. **Methods:** The researchers performed a non-randomized retrospective cohort study which analyzed 206 patients who underwent R0–R1 resection for gastric adenocarcinoma and received either adjuvant CRT (*n* = 107) or CT alone (*n* = 99). A Kaplan–Meier analysis together with Cox regression methods were used to evaluate survival outcomes of patients. Inverse probability of treatment weighting (IPTW) was applied to adjust for baseline differences between groups at the beginning of the study. The median follow-up was 52.0 months. **Results:** The baseline characteristics differed markedly between groups, with CRT patients showing higher rates of T4 tumors (34.6% vs. 22.2%), N3 disease (47.7% vs. 26.3%), vascular invasion (72.9% vs. 50.5%), and R1 resection (10.3% vs. 1.0%). Unadjusted survival favored CT alone (median DFS 81.7 vs. 103.9 months; median OS 86.2 months vs. not reached). These differences lost significance after IPTW adjustment (DFS: HR 1.18, *p* = 0.428; OS: HR 1.24, *p* = 0.336). T3–T4 stage, N2–N3 nodal status, vascular invasion, and positive margins emerged as independent prognostic factors. Subgroup analyses revealed no treatment interactions (all *p* > 0.05). **Conclusions:** The research used a retrospective study design which showed substantial differences between treatment groups at the beginning of the study. The survival results that showed better outcomes for CT alone became attenuated after the researchers applied propensity score adjustment to adjust for confounding from treatment selection. The study established that advanced T–N stage, vascular invasion, and positive margins were identified as independent prognostic factors. The research results are hypothesis-generating and require randomized controlled trials to establish the exact difference in effectiveness between different treatments.

## 1. Introduction

Stomach cancer ranks as the second or third deadliest cancer worldwide because it resulted in 1 million new cases and 770,000 to 783,000 deaths during 2020 [1,2]. The disease is spread distinctly across different geographic areas, with East Asia, Central-Eastern Europe, and South America experiencing high incidence rates but Western nations having lower rates [3,4]. More than 90% of cases exhibit adenocarcinoma histology and are mostly diagnosed at an advanced stage [1]. There are significant differences between Eastern and Western countries in terms of both tumor biology and treatment approaches; intestinal-type and distally located tumors are more common in the East, while proximally located tumors and signet ring cell histology are more prevalent in the West [5]. Surgical resection serves as the primary curative treatment but stands alone as an insufficient solution for locally advanced disease because of its low success rate in preventing both local and distant cancer recurrence [6,7].

Research continues to debate how adjuvant radiotherapy helps gastric cancer patients survive when used with chemotherapy [8]. The ARTIST and CRITICS phase III trials produced conflicting results because their patient diversity and small participant numbers failed to produce conclusive evidence [1,8]. The medical community needs to determine which patient categories receive the most benefit from radiotherapy treatment, especially for patients with lymph node-positive disease and intestinal-type cancer and those at high risk [6]. The current evidence about long-term results from individualized treatment methods based on molecular subtypes remains scarce and there is not enough research about how biomarkers like MSI status and HER2 expression influence treatment choices [2,5]. The medical community requires additional research to understand how different lymph node dissection methods and adjuvant treatment approaches between Eastern and Western guidelines affect patient results because there is no established standardization [3,4].

The main goal of this research involved identifying independent prognostic factors which would help predict the survival rates of gastric cancer patients who received post-gastrectomy adjuvant treatment. The research had two primary research goals, in which treatment outcomes were evaluated and real-world treatment patterns were analyzed between chemoradiotherapy and chemotherapy alone. The study differentiates between prognostic factors, which affect results independently of therapy and predictive factors, which show how patients will react to different treatments; this observational research method enables researchers to study prognostic factors but does not allow definitive conclusions about predictive factors or treatment effectiveness. The research team studied D2 lymph node dissection patients who received chemoradiotherapy to explore potential differences in treatment responses. Our research goal was to generate descriptive data which would help scientists design future studies to test individualized post-surgery cancer treatments for stomach cancer patients.

## 2. Method

### 2.1. Study Population and Sample

This is a retrospective, non-randomized observational study. Due to the inherent limitations of this design, including treatment selection bias, direct comparison of treatment efficacy between groups should be interpreted with caution. The results between groups show different outcomes that may reflect baseline risk differences, which could have caused these differences, rather than treatment effects. This retrospective cohort study included patients diagnosed with gastric adenocarcinoma who underwent curative resection and received adjuvant therapy at our hospital between January 2010 and December 2020. The power analysis for sample size determination used 5-year survival rates from the literature (57.4% vs. 46.1%) to determine that 103 patients per group would achieve 80% power at a 5% type I error rate (α = 0.05) [9]. The research included 206 participants for the study. The study included patients who had received R0 or R1 resection, had histopathologically confirmed gastric adenocarcinoma, had received adjuvant chemotherapy or chemoradiotherapy, were at least 18 years old, and had an ECOG performance score between 0 and 2. The study excluded patients who had metastatic disease, received neoadjuvant therapy, had additional primary cancers, or had insufficient medical records or follow-up periods under 6 months. The study used AJCC 8th edition TNM classification to define operational variables. The Lauren classification system included intestinal, diffuse, and mixed types, which researchers evaluated separately according to established criteria from the literature [9]. The MSI status came under two classifications according to immunohistochemical protein expression analysis, which identified MSS (microsatellite stability) and MSI-H (high microsatellite instability) [10]. Tumor differentiation was classified into two categories according to WHO criteria: well-to-moderately differentiated (Grade 1–2) and poorly differentiated (Grade 3) [11].

### 2.2. Study Procedures

Data collection was performed retrospectively from the hospital electronic record system and patient files; demographic characteristics, clinical parameters, pathology reports, treatment details, and follow-up information were recorded on standardized data collection forms. Pathological evaluations were performed by experienced gastrointestinal pathologists [12]. Vascular invasion was defined as the presence of tumor cells in blood or lymph vessels, and perineural invasion was defined as the presence of tumor cells in the nerve sheath or within the nerve. Surgical margin status was categorized as R0 (no residual tumor) or R1 (microscopic residual, tumor presence within 1 mm of the surgical margin) according to the College of American Pathologists criteria [12,13]. The follow-up protocol, in accordance with NCCN guidelines, included examinations every 3 months for the first 2 years, every 6 months for the next 3 years, and annually thereafter, along with laboratory tests (complete blood count, biochemistry, tumor markers) and imaging methods (chest and abdominal CT) [12,13]. The median follow-up duration was 52.0 months (IQR: 28.4–84.6) for the entire cohort, calculated using the reverse Kaplan–Meier method. Disease recurrence was confirmed by cross-sectional imaging (CT and/or MRI) and histopathological verification when clinically feasible. The reliability of the data collection forms was ensured by having the data of 10% of randomly selected patients reviewed by two independent researchers, with a concordance rate exceeding 95%.

### 2.3. Intervention Protocol

Patients were divided into two groups based on the adjuvant therapy they received: the adjuvant chemoradiotherapy group (*n* = 107) and the chemotherapy-only group (*n* = 99). Patients in the chemoradiotherapy group received a 45–50.4 Gy dose of radiotherapy with concomitant 5-fluorouracil (425 mg/m^2^/day)- or capecitabine (825 mg/m^2^ twice daily)-based chemotherapy; radiotherapy was administered in daily 1.8–2 Gy fractions over 25–28 fractions using three-dimensional conformal radiotherapy or the intensity-modulated radiotherapy technique.

The chemotherapy-only group received treatment with one of the following protocols: CAPOX/XELOX (patients received capecitabine 1000 mg/m^2^ orally twice daily on days 1–14 and oxaliplatin 130 mg/m^2^ intravenously on day 1 for 8 cycles every 21 days) or FOLFOX (patients received oxaliplatin 85 mg/m^2^ and leucovorin 400 mg/m^2^ and 5-fluorouracil 400 mg/m^2^ bolus followed by 2400 mg/m^2^ over 46 h for 12 cycles every 14 days) or 5-FU/LV (patients received leucovorin 20 mg/m^2^ followed by 5-fluorouracil 425 mg/m^2^ on days 1–5 for 6 cycles every 28 days) or capecitabine monotherapy (1250 mg/m^2^ twice daily on days 1–14 with 21-day cycles for 8 cycles). The chemoradiotherapy group received fluoropyrimidine-based chemotherapy which ran simultaneously with radiation therapy before starting maintenance chemotherapy treatment. The researchers documented all planned treatment cycles together with finished cycles and both treatment success percentages and any changes made to the medication dosages.

D2 lymph node dissection was defined according to the Japanese Gastric Cancer Association guidelines as dissection of the first (perigastric, stations 1–6)- and second-tier (stations 7–12, along the celiac axis, common hepatic artery, and splenic artery) lymph node stations [12,13]. The D2 status emerged from surgical records which showed the complete extent of lymph node removal because the removal of 15 or more lymph nodes functioned as a performance metric but did not establish the D2 classification on its own. The research included 41 patients who had unclear D2 status for general analysis but these patients were omitted from the D2-specific subgroup evaluation. Treatment selection between groups was determined based on the decision of the multidisciplinary tumor board and patient preference; no randomization was performed.

### 2.4. Statistical Analysis

Statistical analyses were performed using SPSS version 26.0 (IBM Corp., Armonk, NY, USA) and R version 4.2.0 (R Foundation for Statistical Computing, Vienna, Austria) software. The study reports continuous data through mean (SD) values while showing categorical data as numbers with corresponding percentages. The Student *t*-test was used to compare continuous variables between groups but the Chi-square test or Fisher’s exact test was used for categorical variables when the expected value was below 5 and cell counts were above 20. The Mann–Whitney U test was used for non-normally distributed continuous variables. The duration between surgical intervention and disease return or patient mortality established disease-free survival (DFS) and overall survival (OS), which measured the period between surgery and patient death or their last documented follow-up. Patients were censored at the date of last follow-up for those without events or at the date of death from non-cancer causes for OS analysis. The Kaplan–Meier method generated survival analysis results which the log-rank test evaluated for intergroup differences while reporting median survival periods with 95% confidence intervals.

The Cox regression analysis performed univariate and multivariate assessments to identify prognostic factors which produced hazard ratios (HR) with corresponding 95% confidence intervals (CI). Variables with *p* < 0.10 in the univariate analysis were included in the multivariate model, and the backward stepwise method was used for variable selection. The researchers constructed a pre-specified multivariable Cox model which included essential clinical variables (age, sex, ECOG PS, pT stage, pN stage, histological grade, vascular invasion, perineural invasion, surgical margin status, and D2 lymphadenectomy) even though these variables did not reach statistical significance in the univariate analysis.

Propensity Score Analysis: The research used inverse probability of treatment weighting (IPTW) to handle confounding variables arising from confounding by indication. The researchers used multivariable logistic regression to calculate chemoradiotherapy receipt propensity scores which included the following pre-specified variables: age, sex, ECOG performance status, pT stage, pN stage, histological grade, Lauren classification, vascular invasion, perineural invasion, D2 lymphadenectomy status, and the number of retrieved lymph nodes. Stabilized weights were used to reduce variance, and balance diagnostics were assessed using standardized mean differences. The study used IPTW-adjusted survival curves to compare the results between different groups through weighted log-rank tests, and IPTW-adjusted Cox regression analyzed treatment effects after adjustment for measured confounders.

Sensitivity Analysis: The research team performed all primary survival analyses again using only patients who achieved R0 resection (*n* = 194), excluding patients who had R1 margin status.

A complete case analysis approach was applied for missing data; patients with missing MSI status and D2 dissection information were excluded from the relevant analyses but included in the other analyses. Subgroup analyses were performed according to age (≤60 vs. >60), gender, ECOG performance status (0–1 vs. 2), pT stage (T1–T2, T3, T4), pN stage (N0, N1–N2, N3), and D2 dissection status and were visualized with forest plots. *p*-values for interaction were calculated to assess effect modification across subgroups; these analyses were considered exploratory given the limited statistical power. The researchers used a two-tailed test for all statistical analyses while setting *p*-value < 0.05 as the threshold for statistical significance.

### 2.5. Ethical Considerations

This study was approved by the ethics committee of Kartal Dr. Lütfi Kırdar City Hospital (Decision No: 2025/0l0.99/l5/27, 30 April 2025). A clinical trial number is not applicable. Due to the retrospective design, it was not necessary to obtain additional informed consent from patients. Personal data were anonymized and coded for analysis to protect patient privacy. Data security was ensured on encrypted computers and in databases accessible only to the study team. The study was conducted in accordance with the principles of the Declaration of Helsinki.

## 3. Results

Of the 206 gastric cancer patients included in the study, 107 received adjuvant chemoradiotherapy and 99 received chemotherapy alone. The mean age of the patients was similar in both groups; it was 59.1 ± 11.4 years in the chemoradiotherapy group and 61.4 ± 11.4 years in the chemotherapy-only group. Male patients constituted the majority in both treatment arms, and there was no significant difference between the groups in terms of gender distribution. The majority of the patients had a good performance status; patients with ECOG performance scores of 0–1 accounted for 72% of the chemoradiotherapy group and 70.7% of the chemotherapy group. The researchers followed-up with the participants for 52.0 months on average (IQR: 28.4–84.6) throughout the study period. The chemoradiotherapy group received longer follow-up care than the chemotherapy-only group because their median follow-up period reached 52.3 months (IQR: 28.4–84.6) compared to 38.2 months (IQR: 22.1–58.4) in the chemotherapy-only group (*p* = 0.008) (Table 1).

The pathological evaluation revealed that adenocarcinoma diagnoses were more common in patients receiving chemotherapy alone (85.9% vs. 72.9%, *p* = 0.014). The ‘Other’ histopathological category included signet ring cell carcinoma (*n* = 31) and mucinous adenocarcinoma (*n* = 12). According to the Lauren classification, intestinal-type tumors were significantly more common in the chemotherapy group (65.7% vs. 48.6%, *p* = 0.027). While 72.9% of the patients in the chemoradiotherapy group had poorly differentiated tumors, this rate was 56.6% in the chemotherapy-only group, and the difference was statistically significant. Advanced T stage (T4) tumors were more common in the chemoradiotherapy group (34.6% vs. 22.2%), while early-stage (T1–T2) tumors were more common in the chemotherapy group. In terms of lymph node involvement, N3 disease was observed at a high rate of 47.7% in the chemoradiotherapy group, while this rate remained at 26.3% in the chemotherapy group (Table 1).

The chemotherapy group underwent D2 lymph node dissection at a significantly higher rate than the other group (61.3% vs. 34.4%, *p* < 0.001). The chemotherapy-only group removed more lymph nodes than the chemoradiotherapy group because their median number of retrieved lymph nodes was 22 (IQR: 15–31) versus 18 (IQR: 12–26), respectively (*p* = 0.038). The research obtained sufficient lymph node samples (≥15 nodes) from 74.7% of the patients who received chemotherapy only but from 63.6% of the patients who received chemoradiotherapy. The rate of vascular invasion was higher in patients who received chemoradiotherapy, at 72.9%, compared to 50.5% in patients who received chemotherapy as a standalone treatment. The chemoradiotherapy group showed higher rates of perineural invasion, at 81.3%, compared to 68.7% in the chemotherapy group. There was also a significant difference between the groups in terms of positive surgical margins; R1 resection was 10.3% in the chemoradiotherapy group and only 1% in the chemotherapy group (Table 1).

The chemotherapy treatment protocols between groups showed substantial variations (*p* < 0.001). The chemotherapy-only group received CAPOX/XELOX as their primary treatment, which accounted for 61.6% of cases while FOLFOX was used to treat 21.2% of the patients and capecitabine monotherapy was used for 10.1% of the patients. The chemoradiotherapy group received CAPOX/XELOX as their primary treatment, which accounted for 42.1% of patients, while FOLFOX was used to treat 21.5% of the patients and 5-FU/LV (FUFA) was used to treat 17.8% of the patients. The chemoradiotherapy group received fluoropyrimidine-based regimens (5-FU/LV) at a higher rate because they followed standard concurrent chemoradiation treatment protocols (Table 1).

The treatment completion rates and dose modifications are summarized in Table 2. The median number of completed chemotherapy cycles for CAPOX/XELOX was 6 (IQR: 5–8) in the chemoradiotherapy group and 7 (IQR: 6–8) in the chemotherapy-only group. Treatment completion as planned was achieved in 66.4% of chemoradiotherapy patients versus 76.8% of chemotherapy-only patients. The research findings showed that chemoradiotherapy patients required dose reductions of their medications during 35.5% of their treatment but patients receiving only chemotherapy needed dose reductions during 24.2% of their treatment (*p* = 0.042). The chemoradiotherapy group received a median radiotherapy dose of 45 Gy (IQR: 45–50.4) through 25 fractions, which resulted in a 91.6% completion rate (Table 2).

The study revealed major differences between the treatment groups at the beginning of the research. The chemoradiotherapy group showed more adverse prognostic factors than the other group because 34.6% had T4 tumors compared to a proportion of 22.2%; 47.7% had N3 tumors compared to a proportion of 26.3%; 72.9% had vascular invasion compared to a proportion of 50.5%; 81.3% had perineural invasion compared to a proportion of 68.7%; and 10.3% had R1 margins compared to a proportion of 1.0%. The chemoradiotherapy group received lower rates of D2 lymphadenectomy treatment at 34.4% compared to 61.3% and their surgeons removed fewer lymph nodes during surgery. The study results show treatment selection bias because patients with dangerous medical characteristics received chemoradiotherapy more frequently in this non-randomized research.

The disease-free survival analysis through univariate evaluation demonstrated that the T3 and T4 stages resulted in a 2.85 times and 4.12 times higher recurrence risk than the T1-T2 stages. The risk of disease recurrence grew steadily with advancing lymph node involvement until N3 disease showed a 3.78 times higher risk than N0 disease. The presence of vascular invasion led to a 1.92 times higher risk of recurrence, perineural invasion resulted in a 1.67 times higher risk, and R1 resection led to a 2.18 times higher risk. The risk of disease recurrence became 1.47 times greater for patients who received chemoradiotherapy as an adjuvant treatment compared to those who received chemotherapy only. In the multivariate analysis, T3 (HR: 2.41) and T4 (HR: 3.45) stages, N2 (HR: 2.18) and N3 (HR: 3.31) lymph node involvement, and the presence of vascular invasion (HR: 1.58) were determined as independent prognostic factors.

In the evaluation of overall survival, advanced T stage was a significant indicator of poor prognosis in the univariate analysis; the risk of death was 4.85 times higher in patients with T4 tumors compared to those with T1–T2 tumors. The risk of mortality was 4.21 times higher in patients with N3 lymph node involvement compared to those with N0. The risk of death was 2.05 times higher in the presence of vascular invasion, 1.75 times higher in the presence of perineural invasion, and 2.42 times higher in R1 resection. The risk of mortality was 1.56 times higher in patients receiving chemoradiotherapy compared to those receiving chemotherapy alone. In the multivariate analysis, T3 (HR: 2.58) and T4 (HR: 3.92) stages, N2 (HR: 2.35) and N3 (HR: 3.65) disease, vascular invasion (HR: 1.68), and R1 resection (HR: 2.08) were identified as independent poor prognostic factors.

**Figure 1:**
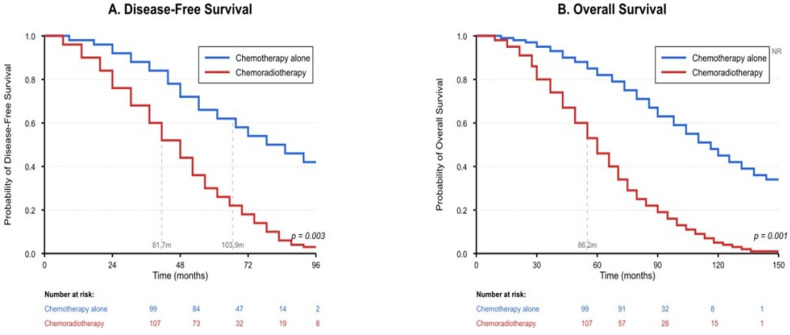
Kaplan–Meier survival curves: (**A**) disease-free survival, (**B**) overall survival. NR, Not Reached.

When the Kaplan–Meier survival curves were examined, the unadjusted analysis showed that although the two groups initially showed similar disease-free survival patterns, an increase in recurrence rates was observed in the chemoradiotherapy group over time. The chemoradiotherapy group achieved a median disease-free survival of 81.7 months while the chemotherapy-only group reached 103.9 months and this difference reached statistical significance at *p* = 0.003. A similar situation was observed in terms of overall survival; the median overall survival was 86.2 months in the chemoradiotherapy group, while this period could not be determined in the chemotherapy-only group, and the 5-year survival rates were found to be 45% and 62%, respectively (*p* = 0.001). The research findings need careful interpretation because the treatment groups showed major differences at the beginning of the study. The Kaplan–Meier survival curve analyses according to the patients’ status of receiving chemoradiotherapy and chemotherapy are shown in Figure 1.

**Figure 2:**
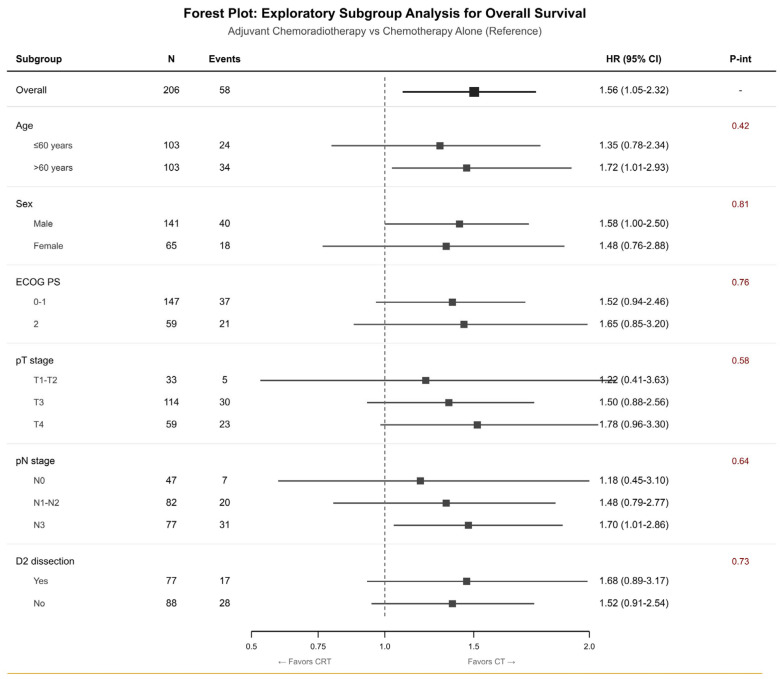
Forest plot of subgroup analysis for overall survival. HR, hazard ratio; CI, confidence interval; P-int, P-interaction.

Exploratory subgroup analyses were performed to assess potential effect modification by baseline characteristics (Figure 2). The hazard ratio point estimates showed that chemotherapy alone performed better than the combination treatment in all analyzed subgroups but the large confidence intervals made it difficult to determine significant differences because they included 1.0 in all subgroups. The analysis revealed no statistically significant interaction effects between variables (all p-interaction values exceeded 0.05: age *p* = 0.42, sex *p* = 0.81, ECOG PS *p* = 0.76, pT stage *p* = 0.58, pN stage *p* = 0.64, D2 dissection *p* = 0.73) which prevented researchers from establishing how treatment outcomes varied between different groups. The similar point estimates between different subgroups indicate that indication confounding affects all groups equally instead of showing actual treatment effectiveness. The research data requires thorough analysis because the study employed observational methods and included few participants in each subgroup and there confounding elements potentially remained.

The research team performed a sensitivity analysis restricted to 194 patients who achieved R0 resection to mitigate potential confounding from surgical margin status. The research included 96 patients who received CRT treatment and 98 patients who received CT treatment. The chemoradiotherapy group developed disease recurrence in 44 patients (45.8%) but the chemotherapy-only group had 15 patients (15.3%) with recurrence (HR 1.86; 95% CI: 1.01–3.41; *p* = 0.045). The study results showed that 40 patients (41.7%) from the chemoradiotherapy group died as compared to 8 patients (8.2%) from the chemotherapy-only group (HR 2.90; 95% CI: 1.34–6.30; *p* = 0.007). The 3-year DFS rates were 58.4% and 82.6%, and 3-year OS rates were 64.2% and 91.8% in the chemoradiotherapy and chemotherapy-only groups, respectively. Despite the exclusion of R1 patients, the research results remained difficult to interpret because the study groups retained substantial baseline differences (Table 3).

**Figure 3:**
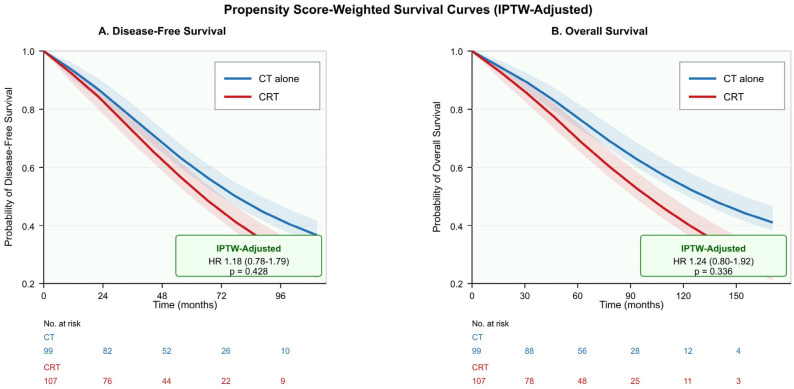
IPTW-adjusted survival curves: (**A**) disease-free survival, (**B**) overall survival. IPTW, inverse probability of treatment weighting.

The analysis used inverse probability of treatment weighting (IPTW) to handle indication-based confounding through propensity scores which were derived from baseline variables that included age, sex, ECOG performance status, pT stage, pN stage, histological grade, Lauren classification, vascular invasion, perineural invasion, D2 lymphadenectomy status, and retrieved lymph node count. After IPTW adjustment, the association between chemoradiotherapy and survival outcomes was substantially attenuated and no longer statistically significant. For disease-free survival, the unadjusted HR of 1.47 (95% CI: 1.01–2.14; *p* = 0.044) was reduced to an adjusted HR of 1.18 (95% CI: 0.78–1.79; *p* = 0.428). For overall survival, the unadjusted HR of 1.56 (95% CI: 1.05–2.32; *p* = 0.028) was reduced to an adjusted HR of 1.24 (95% CI: 0.80–1.92; *p* = 0.336). These findings suggest that the apparent survival disadvantage observed in unadjusted analyses was largely attributable to baseline differences between treatment groups rather than a true harmful effect of chemoradiotherapy (Table 4, Figure 3).

## 4. Discussion

The research examined gastrectomy patients with gastric cancer through their treatment responses and discovery of elements which determine their treatment results. The research established that treatment success depends on three main factors which include surgical extent, tumor biological characteristics, and disease progression stage.

The research results require special attention because treatment assignment created a major selection bias that affected the study results. The data in Table 1 indicate that patients who received chemoradiotherapy treatment started with various negative factors, which included T4 tumors at 34.6% versus 22.2%, N3 nodal involvement at 47.7% versus 26.3%, vascular invasion at 72.9% versus 50.5%, perineural invasion at 81.3% versus 68.7%, and R1 resection margins at 10.3% versus 1.0%. The chemoradiotherapy group received lower rates of D2 lymphadenectomy, at 34.4% compared to 61.3%, and their lymph node retrieval numbers were lower, with a median of 18 compared to 22. The features demonstrate established medical indications for adjuvant radiotherapy which show proper clinical decision-making instead of random treatment selection. Consequently, the observed survival differences between treatment groups are highly likely to reflect confounding by indication rather than true treatment effects.

The research showed that patients treated with chemoradiotherapy displayed more severe tumor behavior than patients who received chemotherapy alone. The vascular invasion rate reached 72.9% in patients who received chemoradiotherapy but only 50.5% in patients who received chemotherapy. Zhang et al. published research in 2023 which showed that stage III disease presented lymphovascular invasion at a rate of 76.91% and T4 tumors showed a substantial increase in this condition [14]. The chemoradiotherapy group showed higher T4 disease rates (34.6% vs. 22.2%) which could explain why vascular invasion occurred more frequently in this group. The perineural invasion rates between our groups (81.3% vs. 68.7%) exceeded the 57.1% overall rate found by Chen et al. but their research demonstrated that perineural invasion was strongly linked to both advanced N stage and poor tumor differentiation [15]. The chemoradiotherapy group showed higher rates of N3 disease (47.7% vs. 26.3%) and poorly differentiated tumors (72.9% vs. 56.6%).

Our D2 lymph node dissection rates showed significant differences between groups; it was performed in 61.3% of the chemotherapy group and 34.4% of the chemoradiotherapy group. In the multicenter study by Yekedüz and colleagues, only patients who underwent D2 dissection were included, and even in this homogeneous group, similar T4 (43% vs. 47%) and N3 (41% vs. 46%) rates were reported in the chemoradiotherapy and chemotherapy arms [16]. In our study, the higher incidence of more advanced disease in the chemoradiotherapy group, where the D2 dissection rate was low, reflects the heterogeneity in patient selection and, possibly, differences in the surgical approach. Furthermore, in our study, the R1 resection rate was high, at 10.3%, in the chemoradiotherapy group, whereas it was only 1% in the chemotherapy group; this supports the notion that patients in the chemoradiotherapy group had more locally advanced disease.

In the unadjusted analyses, patients who received adjuvant chemoradiotherapy showed inferior survival outcomes compared to patients who received chemotherapy as their sole treatment. The median DFS was 81.7 months in the chemoradiotherapy group and 103.9 months in the chemotherapy group, while the 5-year overall survival rates were 45% and 62%, respectively. The researchers exercised caution when analyzing these results because the study participants showed major differences at baseline. These findings contrast with the results reported in the analysis of the ARTIST study from a radiation oncology perspective; Yu et al. reported that adjuvant radiotherapy reduced the locoregional recurrence rate (26.1% vs. 33.8%) and improved locoregional recurrence-free survival, particularly in lymph node-positive patients [17]. The research study demonstrated that the treatment produced limited effects on disease-free survival and overall survival rates according to the study results.

In our multivariate analysis, the attenuation of the association between chemoradiotherapy and survival (*p* = 0.095 for DFS, *p* = 0.077 for OS) suggests that imbalances in baseline characteristics between groups influenced the results. The IPTW-adjusted analysis results demonstrated that the DFS and OS hazard ratios lost their statistical significance at *p* = 0.428 and *p* = 0.336, respectively, which proved that the survival disadvantage stemmed from indication-based confounding. Kim and colleagues’ ARTIST subgroup analysis showed that chemoradiotherapy did not provide additional benefit in early-stage (IB) disease [18]. In our study, the fact that patients in the chemoradiotherapy group had more advanced stage and aggressive tumor characteristics suggests that the survival difference is related to patient selection and baseline prognostic imbalances rather than a true harmful effect of chemoradiotherapy. Lin and colleagues reported that adjuvant chemotherapy provided a significant survival advantage in patients with a high lymph node ratio (3-year OS 46.6% vs. 21.7%) [19]. In our cohort, the higher prevalence of N3 disease in the chemoradiotherapy group (47.7% vs. 26.3%) may explain the poor outcomes in this group.

The research by Allen and colleagues demonstrated that chemoradiotherapy produced better results than chemotherapy in neoadjuvant therapy because 61% compared to 47% of the patients survived for 5 years [20]. The different treatment approaches between neoadjuvant and adjuvant therapy led to this contradictory finding because radiotherapy provides better tumor reduction and pathological response in neoadjuvant treatment yet systemic therapy proves more crucial for controlling microscopic disease in the adjuvant period. The chemotherapy group achieved better R0 resection rates (99% vs. 89.7%) and performed D2 dissections at higher rates (61.3% vs. 34.4%), which demonstrates that surgical quality is associated with survival outcomes.

The exploratory subgroup analysis demonstrated that chemotherapy as a single treatment produced positive results across all studied groups, although the wide confidence intervals did not achieve statistical significance (all p-interaction > 0.05). The research findings show consistent results for all study groups because the study design likely introduced widespread confounding factors, which attenuated any actual treatment benefits. The results do not allow us to determine which specific groups have different treatment outcomes because there are no substantial interaction effects between groups. The study results showed that the observed differences were not modified by the D2 dissection status because the patients who received D2 dissection had an HR of 1.68 (95% CI: 0.89–3.17, p-interaction = 0.73) and patients without D2 dissection had an HR of 1.52 (95% CI: 0.91–2.54). The research findings need careful interpretation because the study used an observational design, there was limited number of participants in each group, and there were potential residual confounding factors. Zhou et al., in their SEER database analysis, reported that neoadjuvant radiotherapy provided a survival advantage in T3-T4 and lymph node-positive patients (HR 0.79) and that this benefit was particularly pronounced in intestinal-type tumors [21]. The higher prevalence of intestinal-type tumors in the chemotherapy group in our cohort (65.7% vs. 48.6%) represents another baseline imbalance that may contribute to the observed differences.

The research shows that chemoradiotherapy treatment produced inferior outcomes in patients who received D2 dissection (HR 1.68). Grassadonia and colleagues noted that direct adjuvant chemotherapy after D2 dissection, which is standard in Asia, may yield different results compared to adjuvant chemoradiotherapy, which is preferred in North America, but that no approach was found to be superior to the other in direct comparative studies [22]. Our findings do not establish that radiotherapy is harmful in patients undergoing D2 dissection; rather, they highlight the need for randomized trials to address this question definitively. In a study by Gao et al., in early-stage (IB) disease, while adjuvant chemotherapy did not show a survival benefit in the general population, a reduction in mortality was observed in patients with high-risk features such as lymphovascular invasion (HR 0.32) [23]. In our study, the higher rate of vascular invasion in the chemoradiotherapy group (72.9% vs. 50.5%) represents a significant prognostic imbalance between groups.

The lack of a significant difference between neoadjuvant chemotherapy and chemoradiotherapy in Hu et al.’s meta-analysis [24] is consistent with the attenuated effect observed in our adjusted analyses. The survival outcomes of elderly patients (>60 years) show a point estimate of 1.72 (*p*-interaction = 0.42), which does not indicate significant effect modification by age.

The research findings showed that T stage, N stage, vascular invasion, and surgical margin status act as independent prognostic factors that influence patient outcomes. Our hazard ratio for disease-free survival in T4 disease was 3.45, which was lower than the value of 4.96 reported by Li et al. but similar to the value of 3.014 reported by Xiaobin et al. [25,26]. The HR values we found for N3 disease (3.31 for DFS, 3.65 for OS) were almost identical to those reported by Zhu et al. (3.29 for DFS, 3.57 for OS) [27]. The research results are consistent with previous studies that show that gastric cancer patients with advanced T and N stages have a poor prognosis; therefore, these factors should remain the main focus for risk assessment.

Our study results showed that vascular invasion proved to be a significant prognostic factor because it resulted in a 1.58 hazard ratio for disease-free survival and a 1.68 hazard ratio for overall survival. These values were quite similar to the results reported by Xiaobin and colleagues (1.581 for DFS, 1.693 for OS) [26]. Zhu and colleagues also identified vascular invasion as an independent risk factor but reported slightly lower HR values (1.45 for DFS, 1.53 for OS) [27]. The adverse effect of R1 resection was consistent across all studies; our HR values (1.89 for DFS, 2.08 for OS) were consistent with the range of 1.77–2.276 reported in the literature [26,27].

Although perineural invasion was significant in the univariate analysis, it lost its statistical significance in the multivariate analysis (DFS *p* = 0.152, OS *p* = 0.131), paralleling the results of Xiaobin and colleagues; they also reported similar *p*-values (DFS *p* = 0.168, OS *p* = 0.147) [26]. This suggests that the effect of perineural invasion is largely due to its association with other aggressive tumor characteristics. It was also noteworthy that D2 lymph node dissection was not identified as a protective factor in the multivariate analysis. The fact that similar results were obtained in both our study (DFS *p* = 0.087, OS *p* = 0.305) and the study by Xiaobin and colleagues (DFS *p* = 0.095, OS *p* = 0.284) suggests that the survival advantage of D2 dissection may be largely related to patient selection and tumor biology [26].

The study by Yuan et al. demonstrated that the HR value for patients with advanced TNM stage (III-IV) reached 2.787 for overall survival and the authors stressed that immune microenvironment markers play a crucial role in determining prognosis [9]. The research supports the implementation of molecular and immunological factors for risk assessment together with traditional pathological indicators. The research established the T stage, N stage, vascular invasion, and margin status as essential factors which should be used for risk assessment to determine which patients need closer monitoring or clinical trial participation.

This research functions as a hypothesis-generating study which presents real-world treatment methods in clinical practice instead of establishing treatment effectiveness. The research results will help scientists develop patient selection methods and predict how study participants will match the population of future randomized controlled trials, but they should not be used to make clinical decisions for patients. Randomized trials need to establish the effectiveness of adjuvant chemoradiotherapy against chemotherapy after gastrectomy surgery through studies which include enough participants and maintain an equal distribution of patients at the beginning of the trial. The non-randomized study design makes it impossible to remove unmeasured variables which could produce residual confounding effects after performing IPTW analysis and all statistical adjustments.

The study’s retrospective design together with treatment selection bias represents the major limitation affecting interpretation. The study contains major baseline differences between groups because doctors made proper treatment choices by giving chemoradiotherapy to patients who needed it most. The study has several additional research constraints because it used data from a single institution, the patients received different chemotherapy treatments, the researchers lacked complete information about the D2 status, and there might have been hidden factors that could have affected the results. Future prospective studies with uniform patient populations will provide a better understanding of treatment effectiveness.

## 5. Conclusions

In this retrospective cohort study, patients receiving adjuvant chemoradiotherapy had significantly worse baseline prognostic features compared to those receiving chemotherapy alone, reflecting the treatment selection bias inherent in clinical practice. While the unadjusted analyses showed inferior survival in the chemoradiotherapy group, the IPTW-adjusted analyses demonstrated a substantial attenuation of this association, suggesting that the observed differences were largely attributable to confounding by indication rather than true treatment effects. The research confirms previous studies by showing that the T stage, N stage, vascular invasion, and margin status serve as independent factors that predict clinical outcomes. The study results function as hypothesis-generating findings which fail to prove the superiority of adjuvant chemoradiotherapy against chemotherapy as a standalone treatment. Randomized controlled trials which enroll patients with balanced baseline characteristics need to be conducted to establish the definitive evidence for this medical condition.

## Figures and Tables

**Table 1:** Baseline clinical, demographic, and pathological characteristics.

Parameter	Adjuvant CRT (*n* = 107)	CT Alone (*n* = 99)	*p*-Value
Age, years (mean ± SD)	59.1 ± 11.4	61.4 ± 11.4	0.142
**Gender, *n* (%)**			0.561
Female	32 (29.9)	33 (33.3)	
Male	75 (70.1)	66 (66.7)	
**ECOG Performance Status, *n* (%)**			0.841
0–1	77 (72.0)	70 (70.7)	
2	30 (28.0)	29 (29.3)	
Histopathology, *n* (%) *			0.014
Adenocarcinoma NOS	78 (72.9)	85 (85.9)	
Other subtypes	29 (27.1)	14 (14.1)	
**MSI Status, *n* (%)**			-
MSS	71 (100)	77 (92.8)	
MSI-H	0 (0)	6 (7.2)	
Unknown	36	16	
**Lauren Classification, *n* (%)**			0.027
Intestinal	52 (48.6)	65 (65.7)	
Diffuse	32 (29.9)	23 (23.2)	
Mixed	23 (21.5)	11 (11.1)	
**Histological Grade, *n* (%)**			0.001
Well/moderately differentiated	29 (27.1)	43 (43.4)	
Poorly differentiated	78 (72.9)	56 (56.6)	
**pT Stage, *n* (%)**			0.001
T1-T2	11 (10.3)	22 (22.2)	
T3	59 (55.1)	55 (55.6)	
T4	37 (34.6)	22 (22.2)	
**pN Stage, *n* (%)**			0.001
N0	16 (15.0)	31 (31.3)	
N1	20 (18.7)	25 (25.3)	
N2	20 (18.7)	17 (17.2)	
N3	51 (47.7)	26 (26.3)	
**Retrieved lymph nodes ^†^**			0.038
Median (IQR)	18 (12–26)	22 (15–31)	
≥15 nodes, *n* (%)	68 (63.6)	74 (74.7)	0.082
**D2 Lymph Node Dissection, *n* (%) ^‡^**			0.001
Performed	31 (34.4)	46 (61.3)	
Not performed	59 (65.6)	29 (38.7)	
Unknown	17	24	
**Vascular Invasion, *n* (%)**			0.001
Absent	29 (27.1)	49 (49.5)	
Present	78 (72.9)	50 (50.5)	
**Perineural Invasion, *n* (%)**			0.023
Absent	20 (18.7)	31 (31.3)	
Present	87 (81.3)	68 (68.7)	
**Surgical Margin, *n* (%)**			0.004
R0 (complete resection)	96 (89.7)	98 (99.0)	
R1 (microscopic residual)	11 (10.3)	1 (1.0)	
**Chemotherapy Regimen, *n* (%) ^§^**			<0.001
CAPOX/XELOX	45 (42.1)	61 (61.6)	
FOLFOX	23 (21.5)	21 (21.2)	
5-FU/LV (FUFA)	19 (17.8)	3 (3.0)	
Capecitabine monotherapy	11 (10.3)	10 (10.1)	
DCF/FLOT	7 (6.5)	2 (2.0)	
Other (CF, ECF)	2 (1.9)	2 (2.0)	

Abbreviations: CRT, chemoradiotherapy; CT, chemotherapy; ECOG, Eastern Cooperative Oncology Group; MSI, microsatellite instability; IQR, interquartile range. * Other subtypes: signet ring cell (*n* = 31), mucinous adenocarcinoma (*n* = 12). ^†^ D2 status based on operative reports; percentages among patients with known status. ^‡^ Baseline imbalances favoring CT group reflect selection bias inherent to this retrospective cohort. ^§^ administered treatment protocols.

**Table 2:** Treatment details and follow-up.

Treatment Parameter	Adjuvant CRT (*n* = 107)	CT Alone (*n* = 99)	*p*-Value
**Planned chemotherapy cycles**			
CAPOX/XELOX (8 cycles)	8	8	-
FOLFOX (12 cycles)	12	12	-
5-FU/LV (6 cycles)	6	6	-
**Completed cycles, median (IQR)**			0.124
CAPOX/XELOX	6 (5–8)	7 (6–8)	
FOLFOX	10 (8–12)	11 (9–12)	
**Treatment completion, *n* (%)**			0.087
Completed as planned	71 (66.4)	76 (76.8)	
Early discontinuation	36 (33.6)	23 (23.2)	
**Dose modifications, *n* (%)**			0.042
Any dose reduction	38 (35.5)	24 (24.2)	
Dose delay (>7 days)	42 (39.3)	31 (31.3)	0.218
**Radiotherapy details (CRT only)**			
Total dose, Gy (median, IQR)	45 (45–50.4)	-	-
Fractions (median)	25 (25–28)	-	-
RT completion rate, *n* (%)	98 (91.6)	-	-
**Follow-up, months**			0.008
Median (IQR)	52.3 (28.4–84.6)	38.2 (22.1–58.4)	
Range	6.2–156.0	6.0–128.5	

Abbreviations: CRT, chemoradiotherapy; CT, chemotherapy; IQR, interquartile range; Gy, Gray; RT, radiotherapy. Note: Median follow-up was calculated using the reverse Kaplan–Meier method.

**Table 3:** Sensitivity analysis: survival outcomes in R0 resection subgroup.

Outcome	CRT (*n* = 96)	CT (*n* = 98)	Unadjusted HR (95% CI)	*p*
**Disease-Free Survival**				
Events, *n* (%)	44 (45.8)	15 (15.3)	1.86 (1.01–3.41)	0.045
Median DFS, months (95% CI)	84.2 (62.4–NR)	NR	-	-
3-year DFS rate, %	58.4	82.6	-	-
**Overall Survival**				
Events (deaths), *n* (%)	40 (41.7)	8 (8.2)	2.90 (1.34–6.30)	0.007
Median OS, months (95% CI)	92.4 (68.2–NR)	NR	-	-
3-year OS rate, %	64.2	91.8	-	-

Abbreviations: CRT, chemoradiotherapy; CT, chemotherapy; HR, hazard ratio; CI, confidence interval; DFS, disease-free survival; OS, overall survival; NR, not reached. Note: This sensitivity analysis excludes patients with R1 resection (*n* = 12) to mitigate potential confounding from surgical margin status. Despite exclusion of R1 patients, significant baseline imbalances persisted between groups.

**Table 4:** Propensity score-weighted analysis (IPTW).

Outcome	Unadjusted HR (95% CI)	*p*	IPTW-Adjusted HR (95% CI)	*p*
Disease-Free Survival	1.47 (1.01–2.14)	0.044	1.18 (0.78–1.79)	0.428
Overall Survival	1.56 (1.05–2.32)	0.028	1.24 (0.80–1.92)	0.336

Abbreviations: HR, hazard ratio; CI, confidence interval; IPTW, inverse probability of treatment weighting. Propensity score model included age, sex, ECOG performance status, pT stage, pN stage, histological grade, Lauren classification, vascular invasion, perineural invasion, D2 lymphadenectomy status, and retrieved lymph node count.

## Data Availability

The datasets generated during and/or analyzed during the current study are not publicly available, but are available from the corresponding author on reasonable request.
